# Tumor-Derived Exosomal miR-620 as a Diagnostic Biomarker in Non-Small-Cell Lung Cancer

**DOI:** 10.1155/2020/6691211

**Published:** 2020-12-01

**Authors:** Youyong Tang, Zhijun Zhang, Xingguo Song, Miao Yu, Limin Niu, Yajing Zhao, Li Wang, Xianrang Song, Li Xie

**Affiliations:** ^1^Department of Clinical Laboratory, Shandong Cancer Hospital and Institute, Shandong First Medical University and Shandong Academy of Medical Sciences, Jinan, China; ^2^Department of Clinical Laboratory, Taian City Central Hospital, Taian, China; ^3^Shandong Provincial Key Laboratory of Radiation Oncology, Shandong Cancer Hospital and Institute, Shandong First Medical University and Shandong Academy of Medical Sciences, Jinan, China; ^4^Department of Clinical Laboratory, Shandong Provincial Third Hospital, Cheeloo College of Medicine, Shandong University, Jinan, China

## Abstract

**Background:**

Evidence has suggested the functional role of exosomal miRNAs in cancer diagnosis. This study aimed to determine whether the serum exosomal biomarkers can improve the diagnosis of patients with non-small-cell lung cancer (NSCLC).

**Materials and Methods:**

The exosomes were extracted from the serum of NSCLC patients (*n* = 235) and healthy donors (*n* = 231) using ultracentrifugation and then were evaluated by using transmission electron microscopy, qNano, and western blotting. The serum exosomal miRNA expression was validated using qPCR.

**Results:**

Exosomal miR-620 was significantly reduced in NSCLC and early-stage NSCLC patients (*P* < 0.0001) when compared to that of healthy controls, with an area under the curve (AUC) of 0.728 and 0.707, respectively. Exosomal miR-620 expression showed an association with drinking (*P*=0.008) and distant metastasis (*P*=0.037). Additionally, the downregulated exosomal miR-620 showed association with chemotherapeutic effect (*P*=0.044).

**Conclusion:**

These findings suggest the serum exosomal miR-620 as a promising diagnostic and prognostic noninvasive biomarker in NSCLC patients.

## 1. Introduction

Approximately 80% of patients suffer from non-small-cell lung cancer (NSCLC), which is the most common subtype and a major cause of morbidity and mortality worldwide [1]. The 5-year overall survival rate of individuals with local NSCLC (stage 1) was as high as 90%. However, most of the NSCLC patients are diagnosed after reaching a more advanced stage, and so these patients have a relatively lower survival rate [2, 3]. Hence, it is imperative to investigate a therapeutic approach for early screening and specific diagnosis of NSCLC patients.

Exosomes are nanoscale vesicles that have a diameter of 30–100 nm and are secreted by almost all types of cells. These were seen across many bodily fluids including urine, saliva, plasma, and malignant secretions [4]. Exosomes translate to protein, miRNA, and mRNA, playing an important role in intercellular communication [5]. Among these, the microRNAs (miRNAs) act as promising molecular markers in diagnosing the tumors [6].

MiRNAs, which are 20–24nt noncoding RNAs, function in posttranscriptional regulation of gene expression in multicellular organisms by directly binding to the 3′untranslated region (3′UTR) of their target mRNA [6]. MiRNAs have been reported to be significant predictive/prognostic biomarkers in different cancers. Studies strongly highlight the potential role of miRNAs in pancreatic ductal adenocarcinoma tumorigenesis, diagnosis, prognosis, and therapy [7]. Several studies have suggested that some specific miRNAs (miR-122, miR-26, and miR-20) have a poor prognosis in patients with hepatocellular carcinoma and are related to poor response to treatment [8]. Moreover, the study showed that aberrant expression of miR-20b, miR-27a, and miR-181a was associated with chemotherapeutic response in gastric cancer suggesting a possible novel therapeutic strategy [9]. More interestingly, miRNAs packed in exosomes are shielded by RNase to prevent degradation when compared to the disposed miRNAs [10]. In addition, the miRNAs packed into exosomes pass through different bodily fluids (i.e., blood and spinal fluid) in a stable cell-free form [11]. Hence, scientists are paying much attention to explore the usefulness of miRNAs wrapped in exosomes, as these might act as possible biomarkers in diagnosis and prognosis of a disease condition [12–14]. Recently, exosomal miRNA has received much attention as a possible diagnostic biomarker in lung cancer [15]. However, to our knowledge, the relationship between exosomal miR-620 expression and the diagnosis and prognostic biomarkers of NSCLC has not yet been clarified.

Hence, in this study, miR-620 was selected for large-scale verification in the serum exosomes of NSCLC patients and healthy donors, establishing a diagnostic group that binds to cytokeratin 19 fragment (CYFRA21-1) and carcinoembryonic antigen (CEA) for the first time to achieve ideal diagnosis of NSCLC with high sensitivity and specificity.

## 2. Materials and Methods

### 2.1. Patients and Healthy Donors

Overall, 235 NSCLC patients and 231 healthy controls from the Shandong Cancer Hospital and Institute, Shandong First Medical University, and Shandong Academy of Medical Sciences (Jinan, China) were recruited between January 2019 and July 2019. All participants provided written informed consent forms for participating in the study. Staging of the tumor was done according to the American Cancer Council's AJCC Cancer Staging Manual (2010). Patients included in this study did not receive any anticancer therapy and had any additional immune, endocrine, or metabolic diseases. Healthy donors included had no other diseases. The characteristics of the patients are listed in Table 1.

### 2.2. Serum Exosomal Isolation

Ultracentrifugation was used to extract the exosomes as described in a previous study [16]. In brief, serum was centrifuged at 3000 g for 10 minutes, again at 10000 g for 30 minutes at 4°C, and finally at 100 000 g for 120 minutes at 4°C (Beckman Coulter) to separate 1 ml supernatant. The pellet was then dissolved in 1 mL phosphate-buffered saline (PBS) or TRIzol reagent to isolate RNA.

### 2.3. Transmission Electron Microscopy (TEM)

The exosomal beads were dripped into the grid with 50 *μ*L of 1% glutaraldehyde, placed for 5 minutes, and then transferred into 100 *μ*L of distilled water. After 2 minutes, the grid was directly transferred to 50 *μ*L uranyl-oxalate solution with a pH of 7 for 5 minutes, and then, the glass dish was covered with sealing film on the ice. The grid was cleaned using distilled water for 7 times for 2 minutes each and then evaluated by JEM-1200EX TEM (JEOL) at 100 kV.

### 2.4. qNano

The size and the particle concentration of exosomes were measured by using TRPS (qNano; Izon Science Ltd). The data were analyzed by using Izon Control Suite software v.3.3.2.2000 (Izon Science Ltd).

### 2.5. Western Blotting Analysis

The extracted protein was separated on 10% SDS-PAGE, transferred onto the PVDF membranes (Millipore), placed in blocking buffer for 2 h, treated with primary antibody at 4°C overnight, and finally treated with secondary antibody at room temperature for 1 h. In addition, the protein was detected on a film through an ECL blot detection reagent (P0018; Beyotime). The main antibodies are as follows: anti-GM130, anti-CD54, and anti-CD9 (CST, America).

### 2.6. RNA Isolation and Real-Time Polymerase Chain Reaction (PCR)

The total RNA of serum exosomes was extracted using TRIzol reagent (Van Allen Way) and then converted to cDNA using the Mix-X miRNA First-Strand Synthesis Kit (Takara Bio). For qPCR, 2 *μ*l cDNA was mixed with the TB-Green Premix Ex Taq II reagent (Takara Bio) and primers in a 20 *μ*l reaction volume. qPCR analysis was done using LC480 (Roche Diagnostics, Germany). U6 served as an internal reference, and the relative expression of serum exosome miR-620 was assessed using the formula ΔCT (Ct_miRNA_-Ct_U6_) [16]. Each sample was assessed in duplicates.

### 2.7. Statistical Analysis

Data were analyzed using SPSS 22.0 (IBM) software and GraphPad Prism 6.0 (GraphPad software). The data were presented as mean with SD or median and interquartile range. The Mann–Whitney *U* test or *t* test was utilized for comparing the two groups. The receiver operating characteristic (ROC) curve analysis was used for discriminating the biomarkers. *P* values of <0.05 were considered to be statistically significant for all comparisons.

## 3. Results

### 3.1. Characterization of Serum Exosomes

**Figure 1:**
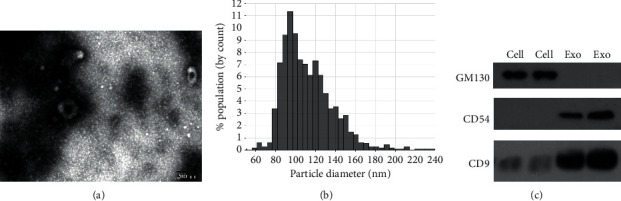
Characterization of serum exosomes. (a) TEM images indicating the characteristic data of exosomes with a diameter of 50–150 nm in NSCLC patients. (b) The exosomes with a size range of 50–150 nm were analyzed by the qNano system. (c) The exosomal protein markers, such as CD54, CD9, and GM130, were detected in the serum by western blotting analysis.

Serum exosomes isolated from NSCLC patients and healthy donors were characterized by TEM, qNano, and western blotting analysis. The results showed typical oval-shaped vesicles under TEM (Figure 1(a)). Correspondingly, the qNano analysis revealed that the diameters of the majority of exosomes ranged between 50 and 150 nm (Figure 1(b)). Additionally, the exosome protein markers, CD54, and CD9 were found to be upregulated in exosomes but not expressed in whole cell lysates. GM130 (negative control) was expressed in cell extracts and not in the extracted exosomes (Figure 1(c)).

### 3.2. Characterization of Identified Serum Exosomal miR-620

**Figure 2:**
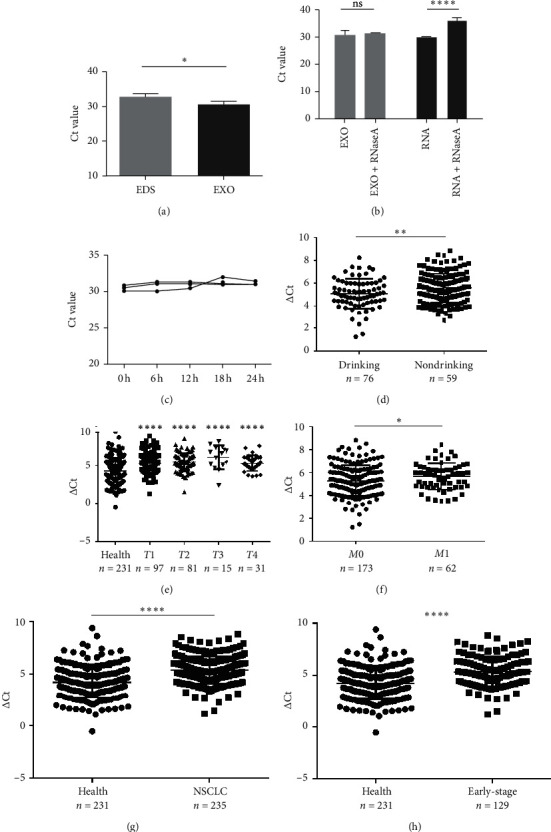
Exosomal miR-620 was decreased in NSCLC significantly. (a) qRT-PCR assessment of miR-620 expression levels from serum exosomes (EXO) and supernatant depleted exosomes (EDS). (b) Analysis of the expression level of miRNA-620 in RNase A-treated exosomes or serum. (c) qRT-PCR analysis of exosome miR-620 expression after incubation at room temperature. The serum exosomal miR-620 demonstrated a significant relationship with (d) drinking status, (e) T stage, and (f) distant metastasis. Serum exosomal miR-620 expression in NSCLC (G) or early-stage NSCLC patients (h) and healthy donors were evaluated by the qRT-PCR assay (^*∗*^*P* < 0.05, ^*∗∗*^*P* < 0.01,^*∗∗∗∗*^*P* < 0.0001, and ns: not significant).

The data of NSCLC patients for miRNA profiling have been described previously [17], in which miR-620 was selected for our next research due to its obvious change in expression. To prove the inclusion of serum miRNA in the exosomes, the expression of miR-620 in exosome-depleted supernatant (EDS) and exosomes was determined. Indeed, miR-620 expression in exosomes showed a significant increase when compared to EDS (Figure 2(a)). Moreover, the stability of exosomal miRNA indicated that miR-620 expression in exosomes showed no change after RNase A treatment (Figure 2(b)). Briefly, these results suggested that miR-620 mainly existed in the exosomes, protecting miRNAs against degradation by RNases. Besides, after leaving the exosomes at room temperature for 0, 6, 12, 18, and 24 hours, no significant changes were observed in the expression of miR-620 (Figure 2(c)).

### 3.3. Exosomal miR-620 Was Decreased in NSCLC Significantly

Next, validation of exosomal miR-620 was done by RT-qPCR in independent serum samples obtained from 235 NSCLC patients and 231 healthy donors. The relationship between exosomal miR-620 expression and the clinical factors is presented in Table 1. Exosomal miR-620 expression showed association with drinking (*P*=0.008, Figure 2(d)) but not with age, gender, smoking history, and pathology (*P* > 0.05). Moreover, the relationship of exosomal miR-620 expression levels was further assessed with TNM stages. Our results revealed that the exosomal miR-620 expression levels showed significant differences in the *T* stage and distant metastasis (Figures 2(e) and 2(f)) but no relation with lymph node metastasis (data not shown).

Next, differential miR-620 expression was identified between NSCLC patients and healthy donors. As depicted in Figures 2(g) and 2(h), the serum exosomal miR-620 expression was significantly downregulated in NSCLC patients (*P* < 0.0001) as well as in early-stage NSCLC when compared with that in healthy groups. This indicated that exosomal miR‐620 acts as a potentially promising NSCLC diagnostic biomarker and predictor of NSCLC metastasis.

### 3.4. Serum Exosomal miR-620 May Serve as NSCLC Diagnostic Marker

**Figure 3:**
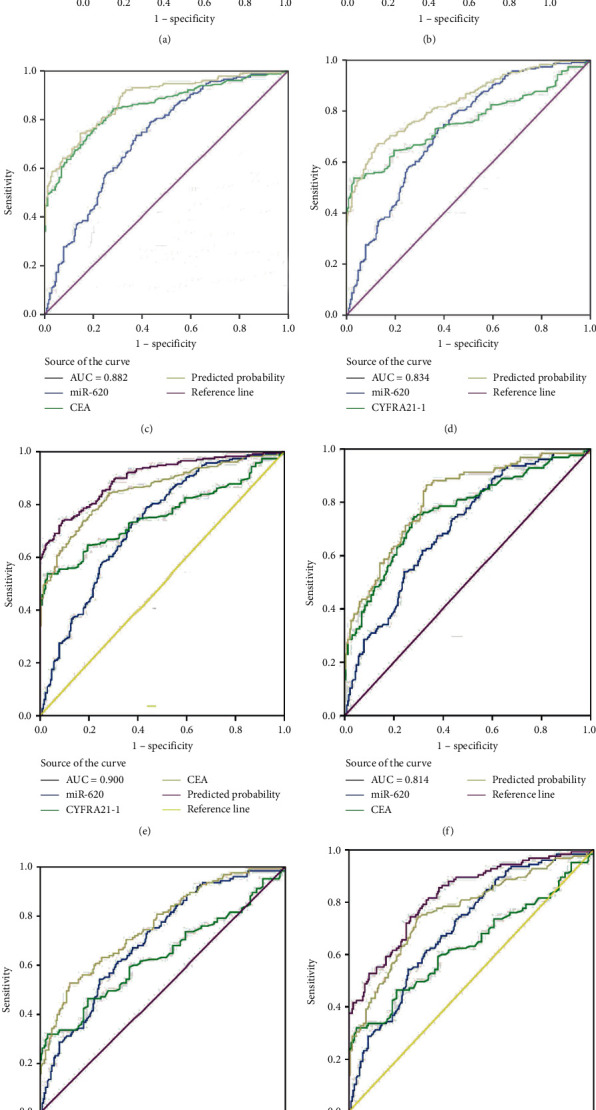
Exosomal miR-620 acts as a diagnostic marker in NSCLC patients. The AUC of serum exosomal miR-620 in patients with NSCLC or early-stage NSCLC and healthy donors was 0.728 (a) or 0.707 (b), respectively. The AUC of serum exosome miR-620 combined with CEA or CYFRA21-1 in NSCLC patients and healthy donors was 0.882 (c) or 0.834 (d), respectively. (e) The AUC of serum exosomal miR-620 in combination with CEA and CYFRA21-1 was 0.900 in NSCLC patients and healthy donors. The AUC of serum exosome miR-620 in combination with CEA or CYFRA21-1 in early NSCLC patients and healthy donors was 0.814 (f) or 0.761 (g), respectively. (h) The AUC of serum exosomal miR-620 in combination with CEA and CYFRA21-1 was 0.824 in early-stage NSCLC patients as well as healthy donors.

To evaluate the diagnostic capacity of exosomal miR-620, the AUC was calculated. Exosomal miR-620 had an AUC of 0.728 with a sensitivity of 74% and specificity of 62.3% or 0.707 with a sensitivity of 62.8% and a specificity of 68.4% when compared between healthy donors and patients with NSCLC or early NSCLC (Figures 3(a) and 3(b)), suggesting its role as a biomarker in diagnosis/early diagnosis of NSCLC.

Moreover, miR-620 with CEA (carcinoembryonic antigen) was combined to facilitate diagnostic efficiency. As shown in Figure 3(c), miR-620 has elevated the AUC of CEA significantly from 0.853 to 0.882. Similarly, the combination of miR-620 with CYFRA21-1 led to an improvement in the diagnosis of NSCLC, as the AUC was elevated from 0.757 to 0.834 (Figure 3(d)). To obtain an improved diagnostic capacity, the exosomal miR-620 with CEA and CYFRA21-1 were merged, leading to a great improvement in the diagnostic ability of NSCLC. The AUC value was 0.900, and the sensitivity was 73.8%, while the specificity was shown to be 90.9% (Figure 3(e)).

Consecutively, the combination of exosomal miR-620 with CEA or CYFRA21-1 showed a significant improvement in the diagnostic ability of early-stage NSCLC (AUC = 0.814 and 0.761, respectively) (Figures 3(f) and 3(g)). Next, the diagnostic ability of the combination of miR-620, CEA, and CYFRA21-1 in early-stage NSCLC patients was assessed. The AUC was 0.824 with a sensitivity of 74.4% and specificity of 74.9% (Figure 3(h)). These results enhanced our observation that exosomal miR-620 acts as a potential biomarker in diagnosis/early diagnosis of NSCLC.

### 3.5. Role of Exosomal miR-620 in Chemotherapeutic Response Assessment

**Figure 4:**
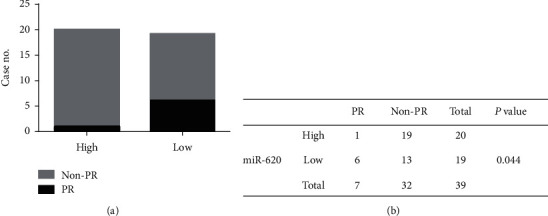
Role of exosomal miR-620 in chemotherapeutic effect prediction.

Additionally, the relationship between serum exosomal miR-620 expression levels and efficacy of chemotherapy was evaluated. Overall, 39 NSCLC patients who received first-line chemotherapy were enrolled in this study. The ratio of patients who experienced a complete response (CR) and partial response (PR) is defined as the remission rate (RR). The proportion of patients with the best response to CR, PR, and stable disease (SD) is designated as the disease control rate (DCR) [18]. The RR of first-line chemotherapy in exosomal miR-620 was 17.9%. Besides, the DCR of first-line chemotherapy in exosomal miR-620 was 79.5% (as shown in Table 2). Furthermore, these patients were separated into PR and non-PR groups. Non-PR included patients with SD and progressive disease (PD). This data suggested that exosomal miR-620 expression was reduced in the non-PR group when compared to that in the PR group (*P*=0.044, Figures 4(a) and 4(b)), demonstrating that exosomal miR-620 might act as a promising biomarker in predicting the chemotherapeutic effect.

## 4. Discussion

Despite the continuous improvement of treatment strategies, NSCLC is still prone to relapse and mortality. Therefore, it is necessary to urgently identify sensitive and specific biomarkers for identifying patients with NSCLC at the early stage. In our study, the exosomal miR-620 expression levels were statistically decreased in patients with NSCLC or early NSCLC, possessing relatively high diagnostic efficiency, with an AUC of 0.728. This indicates that the serum exosomal miR-620 has the potential to act as a biomarker in diagnosing, especially early diagnosis of NSCLC.

Several studies have identified the contribution of miRNAs in the occurrence and progression of multiple cancer types, which thus provides new strategies for tumor treatment, such as biomarkers and cancer therapeutic targets [19]. Identification of miRNAs in serum provides potential applications of these as noninvasive biomarkers for identification, monitoring, and prediction of cancer prognosis, including cancers such as colorectal cancer [20], lung cancer [21], and prostate cancer [22]. Many research studies have also indicated that miR-620 was involved in various biological processes, and aberrant expression of miR-620 showed association with varied cancer types [23, 24]. It has been reported that miR-620 is capable of inhibiting hepatocellular growth and tumorigenesis by blocking the elevation of alpha-fetoprotein [25] and participating in the biological process of colorectal cancer [26], as well as promoting tumor radio resistance through 15-hydroxyprostaglandin dehydrogenase (HPGD) [27].

In our study, several evidences were presented to verify that the exosomal miR-620 serves as a potential biomarker in NSCLC patients. Firstly, the exosomal miR-620 showed significant differences between healthy donors and patients with NSCLC or early NSCLC, indicating its role in tumorigenesis. More importantly, ROC curve analysis demonstrated that exosomal miR-620 exerted diagnostic efficiency with an AUC of 0.728 in NSCLC patients when compared to healthy individuals. Moreover, exosomal miR-620 was significantly downregulated in patients with metastatic NSCLC, indicating its potential role in predicting metastasis. Finally, exosomal miR-620 expression showed an association with response to chemotherapy. Therefore, exosomal miR-620 could be utilized as a possible prognostic indicator to determine that NSCLC patients might benefit from chemotherapy.

In clinical circumstances, the use of serum-derived exosomal miRNA as a biomarker has some advantages. Firstly, the serum-derived exosomes are largely secreted from tumor cells. Compared with tumor tissues, the serum-derived exosomes could more accurately and dynamically reflect the status and function of the tumor cells. Secondly, detecting exosomal miRNAs requires only blood samples, which thereby could overcome the spatial diversity of tissue samples and help to monitor the progression of tumors in a timely manner throughout the treatment process. Furthermore, due to the double membrane structure, miRNAs in exosomes are more stable than those in blood.

However, there are many limitations in this study that should be considered carefully. Firstly, the sample size of NSCLC patients is relatively small. In the future, a larger study with more patients might confirm these findings. Secondly, the specific mechanism and specific function of exosomal miR-620 in different stages of NSCLC have not been evaluated.

In conclusion, this study demonstrated that serum exosomal miRNAs might have diagnostic and prognostic values for diagnosing NSCLC, thus providing novel opportunities as a noninvasive technique and treatment strategy.

## Figures and Tables

**Table 1:** Characteristics of NSCLC patients for differentially expressed serum exosomal miR-620.

Characteristics		No. of cases	Median	*P* value
Age (y)	＜62	115	5.4500	0.235
	≥62	120	5.2350	
Gender	Male	141	5.3550	0.361
	Female	94	5.1625	
Smoking	Yes	122	5.3150	0.502
	No	113	5.2800	
Drinking	Yes	76	5.1075	**0.008**
	No	159	5.4100	
Pathology diagnosis	AC	165	5.3906	0.988
	SCC	59	5.2500	
	Other	11	5.3823	
Lymph node metastasis	Yes	106	5.4250	0.233
	No	129	5.2000	
TNM staging	I-II	129	5.2450	0.440
	III-IV	105	5.4100	
Distant metastasis	Yes	62	5.6626	**0.037**
	No	173	5.2000	

AC, adenocarcinoma; SCC, squamous cell carcinoma.

**Table 2:** Response to the first chemotherapy in serum exosomal miR-620.

Gene	N	CR	PR	SD	PD	RR%	DCR%
miR-620	39	0	7	24	8	17.9	79.5

CR: complete response, PR: partial response, SD; stable disease, PD: progressive disease, RR: response rate; DCR: disease control rate.

## Data Availability

The datasets used and analyzed during the current study are available from the corresponding author on reasonable request.
